# Sirt6 inhibition delays the onset of experimental autoimmune encephalomyelitis by reducing dendritic cell migration

**DOI:** 10.1186/s12974-020-01906-1

**Published:** 2020-07-31

**Authors:** Giovanni Ferrara, Andrea Benzi, Laura Sturla, Daniela Marubbi, Davide Frumento, Sonia Spinelli, Elena Abbotto, Federico Ivaldi, Maria von Holtey, Maximilien Murone, Alessio Nencioni, Antonio Uccelli, Santina Bruzzone

**Affiliations:** 1 Ospedale Policlinico San Martino, IRCCS, Largo R. Benzi, 10, 16132 Genova, Italy; 2 Department of Experimental Medicine (DIMES), University of Genova, Genova, Italy; 3 Department of Neuroscience, Rehabilitation, Ophthalmology, Genetics, Maternal and Child Health (DINOGMI), University of Genova, Genova, Italy; 4 Roche Diagnostics International AG, Rotkreuz, Switzerland; 5 Cellestia Biotech AG, Basel, Switzerland; 6 Department of Internal Medicine and Medical Specialties (DIMI), University of Genova, Genova, Italy

**Keywords:** SIRT6, EAE, Dendritic cells, Migration, Clinically isolated syndrome, MS

## Abstract

**Background:**

Experimental autoimmune encephalomyelitis (EAE) is the most common animal model of multiple sclerosis (MS), a neuroinflammatory and demyelinating disease characterized by multifocal perivascular infiltrates of immune cells. Although EAE is predominantly considered a T helper 1-driven autoimmune disease, mounting evidence suggests that activated dendritic cells (DC), which are the bridge between innate and adaptive immunity, also contribute to its pathogenesis. Sirtuin 6 (SIRT6), a NAD^+^-dependent deacetylase involved in genome maintenance and in metabolic homeostasis, regulates DC activation, and its pharmacological inhibition could, therefore, play a role in EAE development.

**Methods:**

EAE was induced in female C57bl/6 mice by MOG35-55 injection. The effect of treatment with a small compound SIRT6 inhibitor, administered according to therapeutic and preventive protocols, was assessed by evaluating the clinical EAE score. SIRT6 inhibition was confirmed by Western blot analysis by assessing the acetylation of histone 3 lysine 9, a known SIRT6 substrate. The expression of DC activation and migration markers was evaluated by FACS in mouse lymph nodes. In addition, the expression of inflammatory and anti-inflammatory cytokines in the spinal cord were assessed by qPCR. T cell infiltration in spinal cords was evaluated by immunofluorescence imaging. The effect of Sirt6 inhibition on the migration of resting and activated bone marrow-derived dendritic cells was investigated in in vitro chemotaxis assays.

**Results:**

Preventive pharmacological Sirt6 inhibition effectively delayed EAE disease onset through a novel regulatory mechanism, i.e., by reducing the representation of CXCR4-positive and of CXCR4/CCR7-double-positive DC in lymph nodes. The delay in EAE onset correlated with the early downregulation in the expression of CD40 on activated lymph node DC, with increased level of the anti-inflammatory cytokine IL-10, and with a reduced encephalitogenic T cell infiltration in the central nervous system. Consistent with the in vivo data, in vitro pharmacological Sirt6 inhibition in LPS-stimulated, bone marrow-derived DC reduced CCL19/CCL21- and SDF-1-induced DC migration.

**Conclusions:**

Our findings indicate the ability of Sirt6 inhibition to impair DC migration, to downregulate pathogenic T cell inflammatory responses and to delay EAE onset. Therefore, Sirt6 might represent a valuable target for developing novel therapeutic agents for the treatment of early stages of MS, or of other autoimmune disorders.

## Introduction

Sirtuin 6 (SIRT6) belongs to the sirtuin family of proteins, which includes seven members (SIRT1–7). Sirtuins are enzymes that remove acetyl and acyl groups from target proteins by using NAD^+^ as co-substrate [1]. SIRT6 has important roles in physiological and pathological processes, regulating aging, cancer, obesity, insulin resistance, inflammation, and energy metabolism [1]. Regarding immunity and inflammation, our group and others demonstrated that SIRT6 promotes the release of TNFα through its deacetylase activity [2, 3]. Moreover, SIRT6-mediated deacylation of TNFα was reported to enhance the release of this cytokine [4]. In addition to TNFα, SIRT6 also regulates the secretion of other pro-inflammatory cytokines, such as IFNγ and IL8 [3, 5, 6] through the activation of the cation channel TRPM2 [5]. SIRT6 also plays an important role in T lymphocyte biology, since Sirt6KO mice develop lymphopenia [7] and the availability of NAD^+^ for SIRT6 activity is pivotal for the regulation of T cell metabolism during the early and late stages of acute inflammation [8]. SIRT6 also promotes differentiation and maturation of dendritic cells (DC; ref. [9]), which are antigen-presenting cells involved in the initiation of adaptive immune responses. Sirt6KO mice have a reduced representation of DC precursors in their bone marrow; DC from Sirt6KO mice express lower levels of class II MHC, of costimulatory molecules, and of the chemokine receptor CCR7 and are less immunostimulatory than wild-type DC [9].

The lack of SIRT6 inhibitors has so far hampered the study of the effect pharmacological SIRT6 inhibition in animal models. We identified the first small molecules SIRT6 inhibitors, by structure-based in silico compound screens [10]. The use of these inhibitors in in vitro cell systems replicated the biological effects that would be predicted based on SIRT6 role in different cell functions. Our inhibitors reduced TNFα production by phorbol myristate acetate-stimulated cancer cells [10] and by peripheral blood mononuclear cells (PBMC) stimulated with phytohemagglutinin (PHA) or with allogeneic antigen-presenting cells [11]. In addition, they reduced T cell proliferation in response to staphylococcal enterotoxin B (SEB) and to PHA [12]. In line with biological effects regulated by SIRT6, the SIRT6 inhibitors we identified also increased glucose uptake by muscle cells and potentiated the anticancer effects of other chemotherapeutic drugs in cancer cells [10–13]. In addition, a SIRT6 inhibitor with quinazolinedione structure (named **1**) was administered to mice in an in vivo study of SIRT6 inhibition for treating type 2 diabetes [14]. Here, the administration of **1** improved glucose tolerance and other metabolic parameters, such as insulin resistance as well as triglycerides and cholesterol levels [14]. Importantly, **1** was well tolerated with no evidence of clinical toxicity [14].

Based on these studies, here we used the same small molecule SIRT6 inhibitor (**1**) to explore the role of SIRT6 in the development of experimental autoimmune encephalomyelitis (EAE), a prototypical mouse model of autoimmune disorder.

## Materials and methods

### Materials

The SIRT6 inhibitor 2,4-dioxo-*N*-(4-(pyridin-3 yloxy)phenyl)-1,2,3,4-tetrahydroquinazoline-6-sulfonamide (herein named **1**) was obtained from Enamine (Riga, Latvia).

### PK study

Compound **1** was dissolved in 10% kleptose in water at 0.5 mg/mL and pH was adjusted to 4 using 1 N HCl. Mice were injected intraperitoneally with 5 mg/kg body weight of 1. The PK study was carried out as in [12], was approved by the Ethics Committee IACUC (institutional animal care and use committee), and was performed by the company (CRO) Medicilon.

### EAE Induction

Chronic EAE was induced in female C57BL/6 J mice (6–8 weeks of age, weighing 18.5 ± 1.5 g) by subcutaneous injection at two different sites in the right and left flanks with an emulsion (200 μl total) containing 200 μg myelin oligodendrocyte glycoprotein peptide spanning amino acids 35–55 (MOG35–55) (Espikem) in incomplete Freund’s adjuvant (Sigma-Aldrich) supplemented with 600 μg Mycobacterium tuberculosis (strain H37RA; Difco). Mice were injected in the tail vein with 400 ng pertussis toxin (Sigma-Aldrich) in 100 μl of phosphate buffer saline solution (PBS, pH 7.6) immediately and 48 h after the immunization. The mice were scored daily for clinical manifestations of EAE on a scale of 0–5 [15].

All applicable international, national, and/or institutional guidelines for the care and use of animals were followed (Decreto Legislativo 4 March 2014, n. 26, legislative transposition of Directive 2010/63/EU of the European Parliament and of the Council of 22 September 2010 on the protection of animals used for scientific purposes). The research protocol was approved by the Ethical Committee for Animal Experimentation of the IRCCS San Martino hospital, Genoa, Italy, and by the Italian Ministry of Health (project nr, 553/2018-PR).

### Treatment protocol with the SIRT6 inhibitor

The effects of the SIRT6 inhibitor on the severity of EAE was assessed considering two different protocols: **1** was administered following a “preventive” protocol (i.e., administration started at day 0), or following a “therapeutic” protocol (i.e., administration started at disease onset). Mice were randomized in the different experimental groups (11 animals/group). Compound **1** was intraperitoneally injected at two different doses: 30 mg/kg once or twice/day. All the immunized animals developed EAE. Mice were sacrificed at different time points: 7 or 9 days post immunization (dpi), at disease onset in vehicle-treated mice (i.e., at 11–14 dpi) or at 4 days post onset (dpo, i.e., at 15–17 dpi). At sacrifice, blood was immediately collected, transferred into 1.5-ml EDTA coated tubes (Sarstedt), and centrifuged to obtain plasma. Spleen and lymph nodes were collected; cells were dissociated, counted, and subjected to FACS analysis to evaluate DC number (see below). Spinal cords were also collected and immediately frozen or fixed with 4% paraformaldehyde.

### FACS analysis

Cells (collected from spleen and lymph node from **1**- and vehicle-treated mice, see above) were resuspended in 100 μl of FACS buffer (PBS, pH 7.2, containing 0.5% bovine serum albumin) and stained with appropriate conjugated antibodies for 30 min at 4 °C. To assess the migratory markers expression on DC, lymph nodes were collected at 7 dpi and disease onset (11 dpi) and passed through a 70-μm nylon cell strainer (Falcon) to prepare a single-cell suspension and used to optimize staining conditions. Lymph node cells were counted automatically (Countess, Invitrogen), and BV510-positive (Becton–Dickinson) DC (live cells) were gated APC Vio 770-conjugated anti-CD45, Pe Vio-770-conjugated anti-MHCII, and APC-conjugated anti-CD11c antibodies (diluted 1:100, Miltenyi) (see Additional file 1) and antibodies against relevant surface markers, PE-conjugated anti-CCR7, FITC-conjugated anti-CXCR4 (diluted 1:100, Miltenyi), PB-conjugated anti-CD40, or PerCP-conjugated anti-CD86 (diluted 1:100, Biolegend). Data were acquired on a FACS Canto II (Becton–Dickinson) and analyzed using DIVA 6.1 software.

### TNFα determination in plasma

TNFα was evaluated with a commercial ELISA kit (Biolegend, San Diego, CA).

### Immunofluorescence

Spinal cords were fixed overnight in 4% paraformaldehyde in PBS, cryoprotected overnight in 20% sucrose, embedded in Tissue Tek O.C.T., and frozen at − 80 °C. Sections were obtained with a Leica CM3050 S cryostat and permeabilized with 0.1% Triton X-100 (Bio-Rad) in PBS for 30 min. The antibody against CD4 (rat monoclonal anti-mouse CD4 RM4-5 clone (1:100, 550280 BD Pharmingen) was incubated over-night at 4 °C in PBS with 10% goat serum (Sigma-Aldrich) and 0.1% BSA (Sigma-Aldrich). Next, the secondary antibody (goat anti-rat Cy3-conjugated AffiniPure (1:500, 112-165-167 Jackson Immunoresearch Laboratories), was incubated 1 h at room temperature in PBS containing 10% goat serum. Nuclei were stained with Hoechst 33342 (1 μg/ml, Sigma-Aldrich).

### Western blot analysis

Splenocytes were isolated from vehicle and **1**-treated animals at 7 dpi. Primary bone-marrow-derived dendritic cells (BMDDC) were obtained as described below and treated (or not) for 18 h with **1** (50 μM, final concentration). Splenocytes and DC were lysed in 50 mM Tris-HCl (pH 8), 150 mM NaCl, 1 mM EDTA, 1 mM NaF, 10 mM trichostatin A, 10 mM nicotinamide, 0.5 mM DTT, and protease inhibitor cocktail. Lysates (30 μg proteins) were loaded on a 10% polyacrylamide gel and separated by SDS-PAGE, and proteins were transferred to nitrocellulose membranes. Detection was performed with the following primary antibodies: anti-acetylated H3K9 (rabbit polyclonal; Abcam) or anti-vinculin (rabbit monoclonal, Cell Signaling Technology, Danvers, MA). Following incubation with the appropriate secondary antibodies and ECL detection (GE Healthcare, Milan, Italy), band intensity was quantified with the ChemiDoc imaging system (Bio-Rad, Milan, Italy).

### qPCR analyses

RNA extraction from spinal cord was performed using QIAzol Lysis Reagent and TissueLyser instrument (Qiagen); the homogenates were extracted with chloroform, and then RNA was purified using RNeasy Mini Kit (Qiagen) and quantified using a NanoDrop spectrophotometer (Nanodrop Technologies, Wilmington, DE). The cDNA was synthesized by using iScript cDNASynthesis Kit (Bio-Rad Laboratories, Milan, Italy) starting from 1 μg of total RNA. Each sample was assayed in triplicate in a 10-μl amplification reaction, containing 30 ng of cDNA, 0.4 μM of sense and antisense primers, and 10 μl of 2X iQ SYBR Green Supermix Sample (Bio-Rad). The amplification program included 40 cycles of two steps, each cycle including heating to 95 and 60 °C, respectively. To verify the purity of the products, a melting curve was produced after each run. PCR-specific primers were designed through Beacon Designer 2.0 Software (Bio-Rad Laboratories) and were the following: IFNγ, 5′-GGAGGAACTGGCAAAAGGAT-3′ (forward) and 5′-TTCAAGACTTCAAAGAGTCTGAGG-3′ (reverse); IL12, 5′-CCAGGTGTCTTAGCCAGTCC-3′ (forward) and 5′-GCAGTGCAGGAATAATGTTTCA-3′ (reverse); IL10, 5′-TAAGGCTGGCCACACTTGAG-3′ (forward) and 5′-GTTTTCAGGGATGAAGCGGC-3′ (reverse). Values were normalized to murine TBP (TATA Binding Protein) and HPRT1 (Hypoxanthine Phosphoribosyltransferase 1) and β-actin mRNA expression measured using the following specific primers: TBP, 5′-GAAGCTGCGGTACAATTCCAG-3′ (forward) and 5′-CCCCTTGACCCTTCACCAAT-3′ (reverse); HPRT1, 5′-CCCTGGTTAAGCAGTACAGCCCC-3′ (forward) and 5′AGTCTGGCCTGTATCCAACACTTCG-3′ (everse); β-actin, (5′-GGCACCACACCTTCTACAATGAG-3′ (forward) and 5′-GACCAGAGGCATACAGGGACAG-3′ (reverse). Quantitative real-time PCR (qPCR) was performed in an iQ5 real-time PCR detection system (Bio-Rad Laboratories). Statistical analysis of the qPCR was performed using the iQ5 Optical System Software version 1.0 (Bio-Rad Laboratories) based on the 2^−ΔΔCt^ method [16], which calculated the relative change in expression of the target genes, normalized to TBP, HPRT1 and β-actin. The dissociation curve for each amplification was analyzed to confirm absence of unspecific PCR products.

### Generation of bone-marrow-derived dendritic cells

Primary bone-marrow-derived dendritic cells (BMDDC) were obtained as described previously [15]. Briefly, at day 0, bone-marrow cells from mice were flushed from the femur and tibia and passed through a 70-μm nylon cell strainer (Falcon). The cell suspension was seeded in the presence of granulocyte macrophage colony-stimulating factor (20 ng/ml, Miltenyi) and interleukin 4 (10 ng/ml, Biolegend). After 7 days, cells were analyzed by FACS for surface marker CD11c. Purity of CD11c^+^ cells assessed by FACS with APC-conjugated anti-CD11c antibody (Becton–Dickinson) was at least 90%.

### Dendritic cell migration

DC were treated (or not) for 18 h with **1** (50 μM, final concentration) and/or with LPS (0.5 μg/ml, final concentration). DC were resuspended at 8 × 10^5^ cells/ml in chemotaxis buffer (HBSS, PBS, and 5% albumin, 39:16:1). Chemotaxis assays were performed using 96-well ChemoTx system microplates (Neuro Probe, Gaithersburg, MD) with a 5-μm pore size polycarbonate filter. Filters were pre-coated with superfibronectin (1 μg/ml). CCL19 and CCL21 (500 ng/ml) or SDF-1 (300 ng/ml) were added (or not) to the bottom wells. Cell suspensions (25 μl) were then placed on top of the filter and allowed to migrate for 3 h at 37 °C. Counting of transmigrated cells was performed as described previously [17]. Results were expressed as chemotaxis index (number of cells that migrated toward chemoattractant/number of cells that migrated toward medium).

## Results

### SIRT6 inhibition delays EAE onset

Given the role for SIRT6 in lymphocyte and dendritic cell activation [3, 9], we evaluated the effect of the SIRT6 inhibitor, compound **1** in a Th1/Th17-driven model of autoimmune disorder of the central nervous system (CNS), such as EAE. In order to investigate the effect of Sirt6 inhibition during the inflammatory phase and to provide the experimental basis for a possible, new therapeutic approach in MS, **1** was administered according to both a “preventive” (i.e., administration before disease onset) and a “therapeutic” (i.e., administration after disease onset) protocol. **1** was administered at the dosage of 30 mg/kg body weight once a day, starting on the day of mouse immunization with MOG35-55 (preventive treatment) or at the moment of disease onset (therapeutic treatment).

**Fig. 1:**
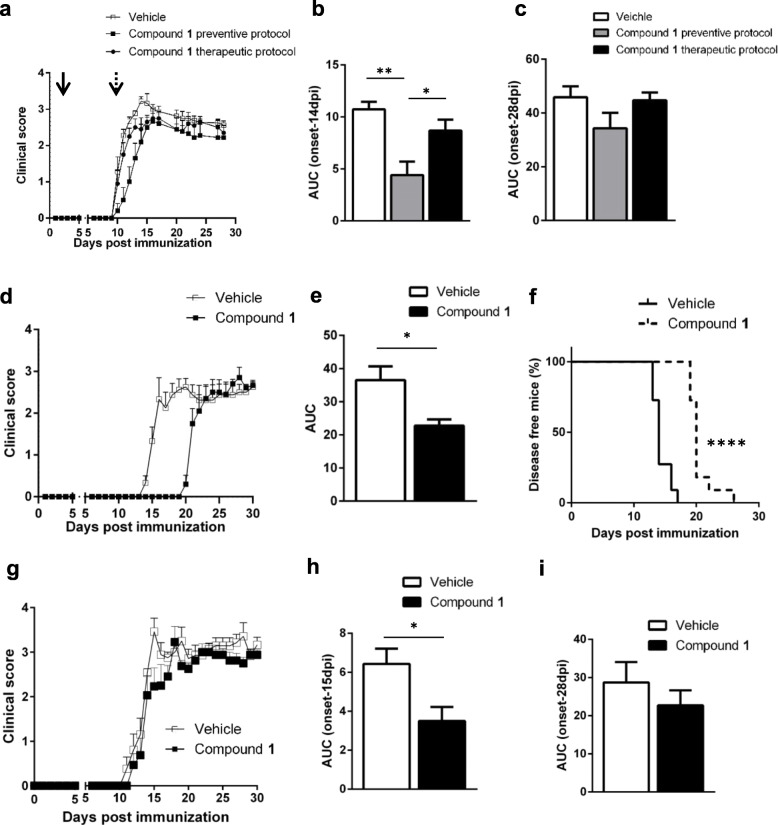
Preventive treatment with compound **1** results in delayed EAE onset. **a** One experiment, representative of two independent experiments, is presented. Each experimental group included 11 mice. In the preventive protocol, **1** was administered intraperitoneally (30 mg/kg, once/day) at 3 dpi (solid arrow); in the therapeutic protocol, **1** was administered at disease onset (10 dpi, dashed arrow). Daily clinical scores are shown as mean ± SEM. **b**, **c** Area under the curve (AUC) of EAE clinical course in mice treated, or not, with **1** (as in panel a) was calculated considering the period from the disease onset to 14 dpi or during the overall EAE course (from onset to 28 dpi). Data are shown as mean ± SEM. **d** EAE course in mice treated with **1** (30 mg/kg, twice/day, 11 mice/group) as preventive protocol from the day of immunization. Daily clinical scores are shown as mean ± SEM. **e**, **f** AUC of EAE clinical score calculated considering the whole EAE course (**e**) and Kaplan-Meier analysis of disease onset (**f**) in mice treated, or not, with **1** (as in panel **d**). **g** EAE course in mice treated with **1** (30 mg/kg, twice/day, 11 mice/group) as therapeutic protocol, starting at disease onset. Daily clinical scores are shown as mean ± SEM. **h**, **i** AUC of EAE clinical course in mice treated, or not, with **1** (as in panel **g**) was calculated considering the period from the disease onset to 15 dpi (**h**) or during the overall EAE course (from onset to 28 dpi, panel **i**). Data are shown as mean ± SEM. *p < 0.05; **p < 0.01; ****p < 0.0001. Data were analyzed by *t* test, except for data in panel **f** (analyzed using the Gehan-Breslow-Wilcoxon test)

As shown in the Fig. 1a, in the preventive protocol, mouse treatment with **1** resulted in a reduction in the neurological impairment (Fig. 1a), and the effect was statistically significant throughout the period between disease onset and day 14 post immunization (14 dpi; Fig. 1b). However, no significant effect of preventive treatment with **1** between day 14 and day 28 was observed (Fig. 1c). When administered according to our therapeutic protocol, **1** failed to show effects on the disease score.

Given the short half-life of **1** (*t*_1/2_ = 0.95 h), a new administration protocol in the preventive treatment was tested, according to which the compound was administered twice daily, starting from the day of immunization. With this schedule, the effect of **1** on disease onset was more pronounced (Fig. 1d, e), with **1** markedly delaying disease onset (Fig. 1f) as calculated by Kaplan-Meier analysis.

When administered twice a day according to the therapeutic protocol, **1** failed to show an impact on disease course, especially in the chronic phase (Fig. 1g–i).

**1** did not show renal or liver toxicity, nor did **1**-treated mice show weight loss, increased frequency of infections, or other clinically detectable adverse events (data not shown and ref. [14]).

### SIRT6 inhibition affects immune cell responses during EAE development

**Fig. 2:**
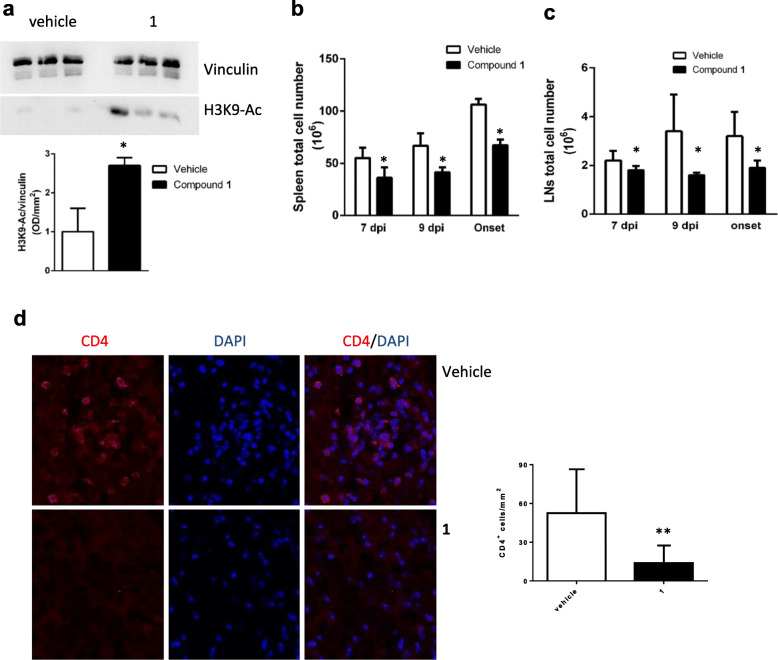
Compound **1** administration in vivo inhibits SIRT6 enzymatic activity and reduces cell number in spleen and lymph node and lymphocyte infiltration in spinal cord. Mice were treated (or not) with **1** (administered twice/day), following the preventive protocol (see the “Materials and methods” section). **a** At 7 dpi, splenocytes were collected and homogenized, and Western blot analyses were performed to evaluate the level of acetylated H3K9 (H3K9-Ac). A representative Western blot analysis is shown, together with the normalized quantification of the band intensity (*n* = 6). **b**, **c** At the indicated time points, the total cell number in spleen (**b**) and in lymph nodes (**c**) was evaluated. Data are expressed as mean ± SD from 6 animals. **d** At 15–17 dpi (i.e., 4 days post onset in vehicle-treated mice), spinal cords were collected from **1**-treated and vehicle-treated animals. Immunofluorescence analyses were performed, upon staining of the infiltrating lymphocytes with an anti-CD4 antibody. Representative images are shown, together with the quantification of at least 10 different images, from 3 animals for each conditions. *p < 0.05, **p < 0.01 compared to the relative control. Data were analyzed by *t* test

In the attempt to identify the mechanism for the observed delay in EAE onset in the animals treated with **1** according to the preventive protocol (administration of **1** twice a day), we analyzed numerous parameters in immune cells that were isolated from spleen and lymph nodes in animals sacrificed on day 7 or on day 9 post immunization (7 dpi, 9 dpi), the latter being two crucial time points of the inflammatory phase, as well as on the day of EAE onset (defined as the moment EAE became clinically detectable in our mice from the control cohort, i.e., 15–17 dpi). Western blot analyses performed on splenocytes obtained at 7 dpi showed that **1** significantly increased the acetylation of histone 3 lysine 9 (H3K9), a known substrate of SIRT6 deacetylase activity (Fig. 2a), thus confirming that i.p. **1** injection reduced SIRT6 enzymatic activity in immune cells in vivo.

As compared to vehicle-treated mice, the total numbers of spleen and of lymph node cells were significantly reduced in animals treated with **1** at 7 and 9 dpi and at disease onset (Fig. 2b, c). Lymphocytes infiltrating the spinal cord were quantified in vehicle- and in **1**-treated mice at 15–17 dpi by immunofluorescence staining of CD4^+^ cells. **1**-treated mice exhibited a significantly lower infiltration with CD4^+^ cells as compared to the control animals, in agreement with the observed difference in clinical score observed at the same time point (Fig. 2d).

### Compound **1** administration reduces the ability of CXCR4^+^ DC to migrate to the afferent lymph nodes in EAE mice

**Fig. 3:**
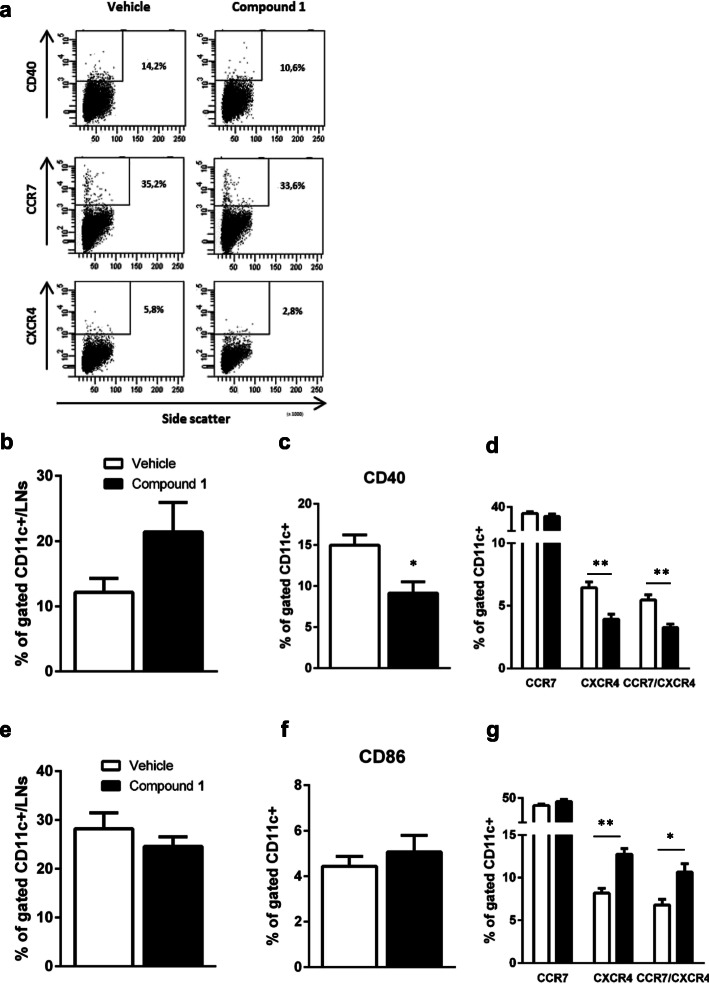
Sirt6 inhibition determines a delayed influx of CXCR4^+^ DC in lymph nodes of EAE-affected mice**.** Mice were treated, or not, following the preventive protocol (i.p. administration of 30 mg/kg **1**, twice/day). **a** One representative plot of CD11c^+^ DC stained CD40, CCR7, and CXCR4 is shown. **b**–**d** DC were obtained from lymph nodes (LNs) at 7 dpi and stained with antibodies against CD11c (**b**), or against CD11c and CD40 (**c**), or against CD11c and CXCR4 and CCR7 (d) and analyzed by FACS. Data are shown as mean ± SEM. **p < 0.01 (*n* = 6 from two independent experiments). **e**–**g** DC were obtained from LNs at 10–13 dpi, i.e., at disease onset in vehicle-treated mice. Cells were stained with antibodies against CD11c (**e**) or against CD11c and CD86 (**f**), or against CD11c and CXCR4 and CCR7 (g). Data are shown as mean ± SEM. *p < 0.05, **p < 0.01 (*n* = 6 from two independent experiments). Data were analyzed by *t* test

We previously reported that Sirt6 plays a role in DC activation [9]. Sirt6KO BMDDC express lower levels of class II MHC molecules and of key chemokine receptors such as CCR2 as compared to WT BMDDC. The triggering of an adaptive immune response requires DC migration from the site of antigen encounter to the draining lymph nodes [18]. Such migration is typically accomplished through the selective expression of chemokine receptors. Immature DC, which reside in or traffic through peripheral tissue, express defined pattern of chemokine receptors, including CXCR4, while not expressing CCR7, which confers responsiveness to inflammatory stimuli and mediates DC migration to the lymph nodes [19, 20]. However, upon exposure to inflammatory signals, DC begin their maturation process, downregulating most chemokine receptors with the exception of CXCR4, while upregulating CCR7 [19, 21]. To define whether Sirt6 inhibition also affects DC function, we quantified the representation of DC in lymph nodes isolated from EAE mice that were treated with or without **1** and also measured CCR7 and CXCR4 expression on them by FACS (Fig. 3a, d, g and Additional file 1).

The percentage of CD11c^+^ DC in lymph nodes from **1**-treated mice was slightly, although not significantly, higher than in vehicle-treated mice at 7 dpi (Fig. 3b). DC activation was significantly reduced by treatment with **1**: specifically, the expression of CD40 (a DC activation marker, which is crucial for the early activation of naïve T cells, ref. [22]) was decreased in **1**-treated mice at 7 dpi (Fig. 3c). Percentages of CXCR4^+^ and of CXCR4^+^/CCR7^+^ double-positive (CD11c^+^) DC were significantly decreased in **1**-treated animals, whereas the percentage of CCR7^+^ CDs cells was only slightly reduced by treatment with **1** (Fig. 3d). At the time of disease onset, in the lymph nodes, the total number of CD11c^+^ DC, as well as the percentage of activated DC (as assessed by the level of CD86, a costimulatory molecule which is crucial for activating T cells, ref. [23]) was not significantly different between **1**-treated and control animals (Fig. 3e, f). However, the percentage of CXCR4^+^ and of CXCR4^+^/CCR7^+^ DC was higher in **1**-treated mice as compared to control animals (Fig. 3g). Altogether, these data suggest that Sirt6 inhibition results in a delay in DC activation and migration: in vehicle-treated mice, DC are activated at 7 dpi, whereas in **1**-treated mice, DC appear to be activated with approximately 5 days of delay (on the day of disease onset in vehicle-treated animals). These findings are in line with the effect of **1** on EAE onset when the compound is used as a preventive treatment (Fig. 1d).

### Pharmacological SIRT6 inhibition hampers DC migration

**Fig. 4:**
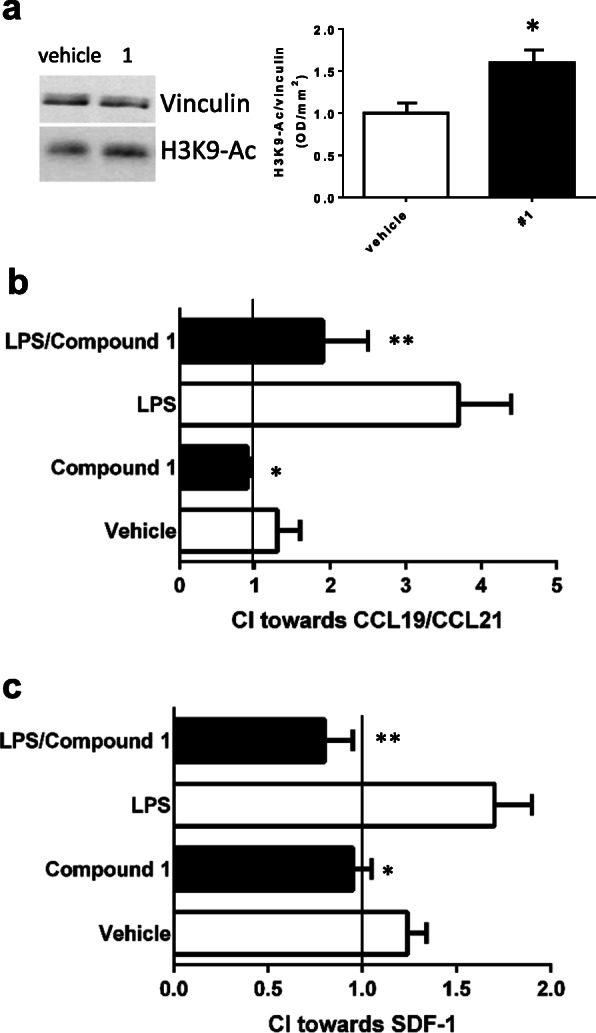
Effect of Sirt6 inhibition on chemotaxis in DC. **a** BMDDC were isolated from wild-type mice, incubated for 18 h in the presence or absence of 50 μM compound **1** and homogenized; Western blot analyses were then performed to evaluate the level of acetylated H3K9 (H3K9-Ac). A representative Western blot analysis is shown, together with the normalized quantification of the band intensity (*n* = 3). **b**, **c** DC were isolated and stimulated (or not) with LPS, in the presence or absence of 50 μM **1** for 18 h. DC were then challenged to migrate toward a medium containing, or not, 500 ng/ml CCL19/CCL21 (**b**) or 300 ng/ml SDF-1 (**c**). Migration was measured in ChemoTx chambers, and the chemotaxis index (CI = number of cells that migrated toward chemoattractant/number of cells that migrated toward medium) was calculated. Results shown are the mean ± SD of three experiments. *p < 0.05; **p < 0.01, compared to the corresponding control. Data were analyzed by *t* test

As mentioned above, a role for SIRT6 in mature DC migration was previously reported based on findings with DC isolated from wild-type and from Sirt6KO mice [9]. Here, we pre-emptively incubated BMDDC from wild-type animals with or without **1** and verified Sirt6 inhibition by monitoring H3K9 acetylation by Western blotting (Fig. 4a). Next, BMDDC generated from wild-type animals and seeded in a 6-well plate, were incubated with or without **1**, and stimulated to mature with LPS. Cells were recovered at the end of the incubation period. The number of viable cells was not significantly affected by the incubation with compound **1** [(3.1 ± 0.5) × 10^5^ and (2.5 ± 0.5) × 10^5^ cells/well were recovered from control and compound 1-treated cells, respectively, *n* = 3]. DC were then challenged to migrate towards a CCL19-CCL21 cocktail (which stimulates CCR7) or towards SDF-1 (which activates CXCR4). As shown in Fig. 4, cell migration was almost completely abolished in the presence of **1**, demonstrating that pharmacological Sirt6 inhibition blocks DC migration. These results are in line with our previous findings in DC from Sirt6 KO mice [9].

### Sirt6 inhibition promotes an anti-inflammatory cytokine profile

**Fig. 5:**
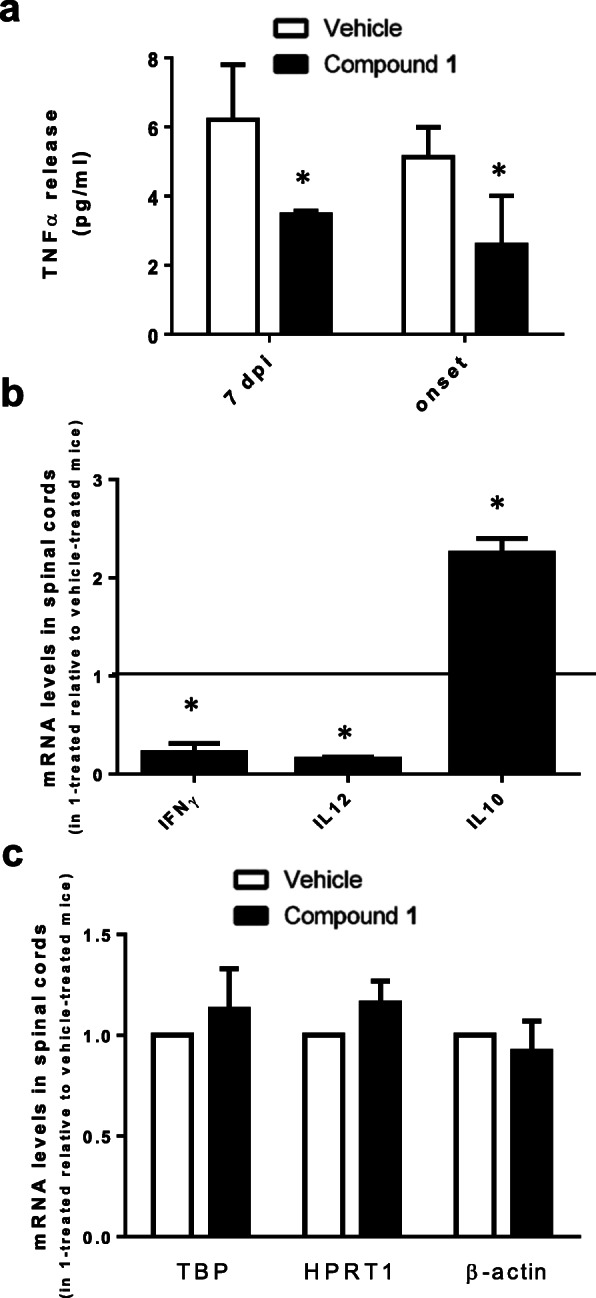
Effect of compound **1** on IFNγ, IL12, and IL10 profile in EAE. Mice were treated (or not) with **1** (administered twice/day), following the preventive protocol (see the “Materials and methods” section). **a** At 7 dpi and at 13–17 dpi (i.e., onset for vehicle-treated mice), mice were sacrificed and TNFα was evaluated in the collected plasma. **b**, **c** Spinal cords were collected at 13–17 dpi (i.e., onset for vehicle-treated mice), and the relative expression of IFNγ, IL12, and IL10 (**b**) and of TBP, HPRT1, and β-actin (used as “housekeeping” genes) (**c**) was analyzed by qPCR analysis. Samples from at least 4 animals were analyzed for each condition. *p < 0.05, compared to controls (data analyzed by *t* test)

Given the role of Sirt6 in regulating TNFα expression and release [2–5], we proceeded to measure TNFα concentration in mouse plasma. The levels of TNFα were significantly decreased in animals treated with the SIRT6 inhibitor, **1**, both at 7 dpi and at 15–17 dpi, i.e., at disease onset in vehicle-treated mice (Fig. 5a). We also evaluated the level of IFNγ and of IL12, two cytokines that promote Th1 immune responses, by qPCR on spinal cords collected at EAE onset and found that their expression was also reduced in mice which were given **1** (Fig. 5b). Finally, the expression of IL10, which has anti-inflammatory effects, was significantly increased in the spinal cord of **1**-treated mice (Fig. 5b). IFNγ, IL12, and IL10 expression values were normalized against the housekeeping TBP and HPRT1 genes, since their expression, as well as the expression of β-actin, were not different in spinal cords from animals treated with **1** or with the vehicle (Fig. 5c). Altogether, these data confirm that the delay in EAE onset via treatment with the Sirt6 inhibitor, **1**, likely reflects a delayed activation of the immune response.

## Discussion

Both cells of the adaptive and of the innate immune system are involved in several deleterious steps of autoimmune responses, including activation of T cells by DC. EAE is a complex model in which the interaction between a variety of immune system components and the CNS leads to pathological features that are very similar to those of MS: inflammation, demyelination, axonal loss, and gliosis [24]. The adjuvant that is used in EAE to stimulate peripheral immune cells contains killed Mycobacterium tuberculosis, which acts as danger signal: both MOG35-55 and the adjuvant are injected subcutaneously to stimulate local DC that subsequently migrate to the draining lymph nodes to stimulate antigen-specific T cells. After successful activation, encephalitogenic T cells migrate into the CNS to cause tissue damage. Thus, EAE is used as a model of cell-mediated organ-specific autoimmunity. Our goal was to determine the effect of pharmacological Sirt6 inhibition in DC in EAE as a model of autoimmune disorders.

SIRT6 plays critical roles in multiple molecular pathways, including DNA repair, telomere maintenance, glycolysis, gluconeogenesis, lipid metabolism, and inflammation [25]. Different attempts to modulate its activity have been investigated as a means to provide therapeutic benefits in different conditions. Mainly in view of its role in DNA repair and in telomere biology and as a tumor suppressor in different cancer type, activation of SIRT6 is postulated to have beneficial effects and to promote healthspan [25]. Nevertheless, the development of SIRT6 inhibitors has also been pursued as these compounds could find application in some cancer types, in which SIRT6 seems to have pro-oncogenic effects (i.e., squamous cell carcinomas, pancreatic cancer, acute myeloid leukemia, and multiple myeloma), such as increasing cell migration, tumor DNA repair, and secretion of pro-angiogenic factors [5, 13, 26, 27]. In addition, given the fact that SIRT6 regulates insulin signaling [28] and the expression of the glucose transporters GLUT1 and GLUT4 [7, 28], we previously tested **1** in a mouse model of type 2 diabetes. Here we found different metabolic parameters to be improved by **1**, including glycemia, insulinemia, triglycerides, and cholesterol, following activation of glucose consumption [14]. In addition, enhancement of glycolysis via SIRT6 inhibition was shown to promote survival of photoreceptor and to preserve vision in a mouse model of retinitis pigmentosa [29]. Concerning neurodegenerative and inflammatory diseases, SIRT6 inhibition was proposed to be protective at least in certain circumstances [30]. For instance, inhibiting SIRT6 was suggested as a promising strategy against Parkinson’s disease, given that SIRT6 has pro-inflammatory effects in this disorder and thereby accelerates its course [30].

As reported in the “Introduction” section, SIRT6 plays a multifaceted role in the regulation of the immunity, which can be summarized as follows: (a) SIRT6 promotes the release of TNFα from different cells (including DC) through different mechanisms [2–4, 9]; (b) SIRT6 promotes the secretion of IFNγ and of IL8 [3, 5, 6]; and (c) SIRT6 enhances DC differentiation and maturation [9].

Thus, based on SIRT6 role in inflammation and in DC, we decided to test the SIRT6 inhibitor, **1**, in the EAE model of autoimmunity according to both a “preventive” and a “therapeutic” protocol. The most interesting result from this study was the delay in EAE onset, which was obtained by inhibiting Sirt6 according to our “preventive” approach. By this approach, we aimed at investigating the effect of Sirt6 inhibition during the early phase of the inflammatory process preceding the overt disease. In line with the studies indicating SIRT6 as a key player in regulating pro-inflammatory responses, our results indicate that SIRT6 inhibition delayed the inflammatory cascade occurring upon immunization and before EAE onset. Specifically, SIRT6 inhibition with **1** reduced the number of splenocytes and of lymph node cells in mice (Fig. 2b, c); blunted the counts of lymphocytes infiltrating the spinal cord (Fig. 2d); reduced the production of the autoimmunity-promoting cytokines, IFNγ and IL12 (Fig. 5); and increased the production of the anti-inflammatory cytokine, IL-10 (Fig. 5). In addition, **1** reduced the percentage of mouse lymph node DC expressing the pro-migratory surface marker, CXCR4 (Fig. 3).

Given the well-established role of peripheral and CNS-resident DC in the development of EAE [31, 32], we propose that the delay in the onset of EAE upon mouse treatment with **1** reflects the effect of Sirt6 inhibition on DC migration, as revealed by the reduced representation of CXCR4-positive and of CXCR4/CCR7-double-positive DC in lymph nodes [33] of **1**-treated animals (Fig. 3) and by the impaired migration of **1**-pre-treated DC in response to chemokines (Fig. 4). The mechanism by which Sirt6 inhibition reduced DC migration *in vivo* might also reflect the fact that TNFα release (as measured by evaluating TNFα in plasma, Fig. 5a) was reduced upon SIRT6 inhibition, which is in line with the reported ability of SIRT6 to regulate TNFα production by different cell types [2–5]. Indeed, TNFα is a crucial cytokine in DC activation and migration [34, 35].

Overall, our results provide a rationale for further exploring SIRT6 inhibition as a strategy to treat MS or other autoimmune disorders by virtue of its effects on the DC compartment. Given the fact that Sirt6 inhibition failed to show an impact on disease course in the therapeutic protocol (where **1** administration was started at disease onset; see Fig. 1 and the “Results” section), SIRT6 is unlikely to represent a viable target for treating the overt MS. Nevertheless, SIRT6 inhibition may be tested during the early stages of an immune-mediated condition to prevent exacerbations and progression of the disease. For instance, the so-called clinically isolated syndrome (CIS) is often the first indicator of MS, with few available therapeutic options to avoid its progression to overt MS [36]. As compared with healthy subjects, patients with CIS exhibit a peripheral blood signature that is characterized by a higher frequency of DC, and this suggest a role of these antigen-presenting cells in CIS conversion to MS [37]. Thus, early treatment of these patients with a SIRT6 inhibitor could conceivably avoid or at least slow the CIS progression to MS, due to the effect exerted on DC migration.

## Conclusions

The present study demonstrates that preventive pharmacological SIRT6 inhibition delayed EAE disease onset, reducing the representation of CXCR4-positive and of CXCR4/CCR7-double-positive DC in lymph nodes. The delay in EAE onset was associated with the early downregulation of CD40 expression on DC and with reduced encephalitogenic T cell infiltration in the CNS. Sirt6 inhibition reduced the production of the autoimmunity-promoting cytokines, IFNγ and IL12, and increased the production of the anti-inflammatory cytokine IL-10. Therefore, Sirt6 might represent a valuable target for developing novel therapeutic agents for the treatment of the early stages of MS or of other autoimmune disorders. The administration of SIRT6 inhibitors should be done with caution, limiting it to defined time windows, taking into account that this enzyme also has a role as a tumor suppressor in certain tissues and that complete SIRT6 depletion can be very detrimental [7, 23]. Nevertheless, as discussed above, growing evidence does point to SIRT6 inhibition as to a promising strategy in defined conditions, including MS.

## Supplementary information

**Additional file 1:. Figure S1.** Gating strategy for flow cytometry analysis of DCs

## Data Availability

The datasets used and/or analyzed during the current study are available from the corresponding author on reasonable request.

## Abbreviations

SIRT6: Sirtuin 6; 2,4-dioxo-N-(4-(pyridin-3 yloxy)phenyl)-1,2,3,4-tetrahydroquinazoline-6-sulfonamide (SIRT6 inhibitor), **1**
DC: dendritic cells
EAE: experimental autoimmune encephalomyelitis
MOG: myelin oligodendrocyte glycoprotein
dpi: days post immunization
dpo: days post onset
LNs: lymph nodes
CXCR4: C-X-C chemokine receptor type 4
CCR7: C-C chemokine receptor type 7
MS: multiple sclerosis
CIS: clinically isolated syndrome
