# Supplementary material for: Sirt6 inhibition delays the onset of experimental autoimmune encephalomyelitis by reducing dendritic cell migration
Source: J Neuroinflammation. 2020 Jul 31;17:228. doi: 10.1186/s12974-020-01906-1 (PMC7393881; doi:10.1186/s12974-020-01906-1)
Supplement: Supplementary file 1 — Additional file 1:. Figure S1. Gating strategy for flow cytometry analysis of DCs [file 12974_2020_1906_MOESM1_ESM.pdf]

Additional data

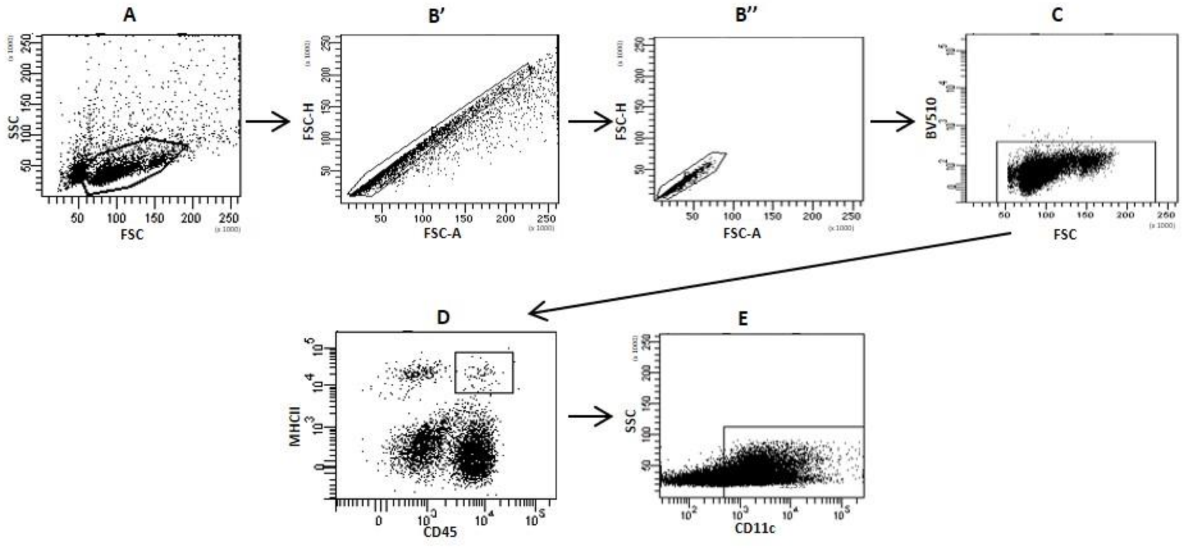

Additional Figure 1. Representative gating strategy for flow cytometry analysis, for the experiments shown in Figure 3. CD11c-positive DC were analyzed by flow cytometry from the lymph nodes. Immune cells were selected by morphological parameters based on forward and side scatter (A), doublets were then excluded (B',B''). BV510 negative (live) cells were selected (C) and then gated for CD45 and MHCII (D). This double positive cell population was then assessed by the specific DC marker CD11c (E).
